# Crosstalk between G-Quadruplexes and Dnmt3a-Mediated Methylation of the *c-MYC* Oncogene Promoter

**DOI:** 10.3390/ijms25010045

**Published:** 2023-12-19

**Authors:** Alexander V. Sergeev, Andrei G. Loiko, Adelya I. Genatullina, Alexander S. Petrov, Elena A. Kubareva, Nina G. Dolinnaya, Elizaveta S. Gromova

**Affiliations:** 1 Department of Chemistry, Lomonosov Moscow State University, Leninskie Gory 1, 119991 Moscow, Russia; avsergeev@belozersky.msu.ru (A.V.S.); andrewloykochem@gmail.com (A.G.L.); genatullinaadela@gmail.com (A.I.G.); asp2109@yandex.ru (A.S.P.); dolinnaya@hotmail.com (N.G.D.); gromova@belozersky.msu.ru (E.S.G.); 2 Belozersky Institute of Physico-Chemical Biology, Lomonosov Moscow State University, Leninskie Gory 1, 119991 Moscow, Russia

**Keywords:** G-quadruplexes, *c-MYC* promoter, DNA methylation, Dnmt3a, DNA–protein interaction

## Abstract

The methylation of cytosines at CpG sites in DNA, carried out de novo by DNA methyltransferase Dnmt3a, is a basic epigenetic modification involved in gene regulation and genome stability. Aberrant CpG methylation in gene promoters leads to oncogenesis. In oncogene promoters, CpG sites often colocalize with guanine-rich sequences capable of folding into G-quadruplexes (G4s). Our in vitro study aimed to investigate how parallel G4s formed by a sequence derived from the *c-MYC* oncogene promoter region affect the activity of the Dnmt3a catalytic domain (Dnmt3a-CD). For this purpose, we designed synthetic oligonucleotide constructs: a *c-MYC* G4-forming oligonucleotide and linear double-stranded DNA containing an embedded stable extrahelical *c-MYC* G4. The topology and thermal stability of G4 structures in these DNA models were analyzed using physicochemical techniques. We showed that Dnmt3a-CD specifically binds to an oligonucleotide containing *c-MYC* G4, resulting in inhibition of its methylation activity. *c-MYC* G4 formation in a double-stranded context significantly reduces Dnmt3a-CD-induced methylation of a CpG site located in close proximity to the quadruplex structure; this effect depends on the distance between the non-canonical structure and the specific CpG site. One would expect DNA hypomethylation near the G4 structure, while regions distant from this non-canonical form would maintain a regular pattern of high methylation levels. We hypothesize that the G4 structure sequesters the Dnmt3a-CD and impedes its proper binding to B-DNA, resulting in hypomethylation and activation of *c-MYC* transcription.

## 1. Introduction

In addition to the double-stranded B-form, genomic DNA can fold into various sequence-dependent, non-canonical structures. Among them, G-quadruplexes (G4) are the most studied and biologically significant form of nucleic acids.

Endogenous G4s are formed via intramolecular interactions of G-rich nucleic acids whose sequences contain at least four tandem G-tracts of consecutive guanosines separated by quasi-random nucleotide residues [1,2]. G4 structures are stabilized by two or three stacked G-tetrads, planar arrangements of four guanines from different G-tracts connected via Hoogsteen base pairing. Nucleotide sequences between G-tracts can form G4 loops that play an important role in determining quadruplex topology and stability. The G4 core is further stabilized by the coordination of guanine O6 with monovalent cations, predominantly K^+^, in the central cavity [3,4]. In vitro, it was shown that G4 structures can have a wide range of folds, differing in the orientation of the G-tracts, adopting parallel, antiparallel, or mixed (3 + 1) topologies, the type of loops and their length, the number of G-tetrads, as well as the local structural parameters; some of the G4s are highly dynamic, while others adopt only one conformation. More than 700,000 G4 motifs have been identified in the human genome [5]. Bioinformatics analysis has shown that G4 motifs are frequently clustered in the promoter regions of many oncogenes and genes involved in growth control [6,7,8], replication origins, untranslated exon regions, telomeric DNA, and micro(mini)satellite repeats [9,10,11]. The formation of G4 structures in promoter regions in vivo occurs in competition with the maintenance of the B-DNA double helix and is regulated by various opposing driving forces. The factors modulating G4 folding and stability in vivo include: nucleosome-free chromatin regions, negative DNA supercoiling, biological processes causing local B-DNA unwinding (DNA replication, transcription, DNA recombination, DNA repair, etc.), G4-stabilizing and destabilizing cellular proteins, and exclusion of C-rich strands from the B-DNA–G4 equilibrium due to the formation of a stable i-motif or co-transcriptional R-loop [12]. 

Currently, G4s are considered novel regulatory elements that are involved in key genome functions such as transcription, telomere maintenance, DNA replication, and repair [13,14]. On the other hand, G4 formation causes accidental genome and epigenome instability, associated with carcinogenesis. In recent years, the detrimental effects of G4s on genome integrity and their potential role in regulating DNA repair machinery have become the subject of intense research [12]. In addition, numerous connections of G4 structures to cancer biology have been proposed. Thus, G4 in promoters of oncogenes performs regulatory functions, acting as a transcriptional silencer element that can be targeted with potential anticancer drugs [14,15,16,17].

Much less studied is the role of promoter G4s in another key biological process: the methylation of cytosine residues at CpG sites. These basic epigenetic DNA modifications are involved in the regulation of gene expression, maintenance of genomic stability, aging, etc. In mammals, de novo DNA methylation, i.e., the implementation of the methylation pattern (specific alternation of methylated and unmethylated CpG sites) is performed by DNA methyltransferase (MTase) Dnmt3a [7,8]. The maintenance of the methylation pattern during DNA replication is carried out by the Dnmt1 MTase [18]. Despite the primary role of Dnmt1 in maintaining methylation, cooperation between Dnmt3a and Dnmt1 has been observed in some cases, particularly in embryonic stem cells. The promoter regions of actively transcribed genes containing CpG islands are usually unmethylated. In contrast, the promoters of oncogenes, unlike tumor suppressor genes, are hypermethylated. Methylated cytosines are potentially mutagenic due to their spontaneous deamination, leading to C > T point substitutions. Therefore, the presence of 5-methyl-2′-deoxycytosine residues in oncogene promoters can affect the DNA repair pathways and lead to somatic “driver mutations”. Thus, the level of methylation in regulatory regions of the mammalian genome is directly related to the progression of several types of cancers [19]. Evidence of the participation of promoter G4s in transcriptional regulation, as well as the dependence of the transcription level on the methylation status of CpG islands in oncogene promoters, makes it relevant to analyze the G4 impact on the DNA methylation level. However, there are only a few studies on the involvement of G4 structures in the functioning of DNA methylation machinery [13,20]. Nevertheless, studies based on whole-genome sequencing data identified a correlation between CpG methylation levels and G4 formation, namely, hypomethylation of the genome in regions enriched in G4 motifs [21,22]. The G4 formation in the promoters of imprinted genes was revealed [23]. Using surface plasmon resonance spectroscopy, the authors reported the high binding affinity of recombinant human MTases to quadruplex structures formed in synthetic oligonucleotides containing G4 motifs of the corresponding promoter regions. The binding affinity of the enzyme to G4 has been shown to be comparable to that of other cellular proteins that specifically interact with G4s. Recently, the interplay between G4 formation and DNA methylation levels has been confirmed [24]. Using sequence analysis, it was shown that the majority of G4 motifs found in the human genome are localized in regions of unmethylated CpG islands. The authors also discovered the co-localization of G4 motifs and the binding sites of maintenance MTase DNMT1 and proposed a mechanism for protecting CpG islands from methylation with G4 structures that effectively bind and inhibit DNMT1. However, the G4 effect was characterized indirectly by inhibiting methylation of a standard substrate with G4-forming oligonucleotides. The G4 impact on de novo methylation by Dnmt3a has not been studied at all.

Given the crosstalk between DNA methylation status and G4 formation in oncogene promoters, the main goal of our work was to evaluate the effect of this non-canonical DNA structure on Dnmt3a function. In our study, we selected the G4 motif of the *c-MYC* promoter. This gene product is a major oncogenic driver in cancer involved in the regulation of cellular proliferation, differentiation, and apoptosis [25]. Aberrant expression of the *c-MYC* oncogene (usually *c-MYC* hyperexpression) leads to the malignant transformation of cells. The 27-nt G-rich region of the *c-MYC* promoter (Pu27) located in the nuclease hypersensitive element (NHE) III_1_ has the ability to fold into intramolecular parallel G4 structures [6,17,26]. Five tandem G-tracts (underlined) in this sequence, 5′-TGGGGAGGGTGGGGAGGGTGGGGAAGG-3′, lead to the potential formation of multiple G4s [8]; the resulting G4 structures differ in the positions of the four G-tracts involved in their formation and, consequently, in their conformational features and thermodynamic stability. Nevertheless, a parallel arrangement of G-tracts characteristic of promoter G4 structures is realized in this mixture of G4s, which act as a transcriptional repressor. 

In our work, we aimed to investigate how parallel G4s formed by a sequence derived from the *c-MYC* oncogene promoter region affect the activity of the murine Dnmt3a catalytic domain (Dnmt3a-CD) in a double-stranded context. Using this DNA model, we evaluated the role of the distance between the G4 structure and the analyzed CpG site on its methylation. Furthermore, we examined the influence of isolated *c-MYC* G4 on the Dnmt3a-CD function.

## 2. Results

### 2.1. Synthetic G4-Containing DNA and Control Single- and Double-Stranded Oligonucleotides

**Figure 1:**
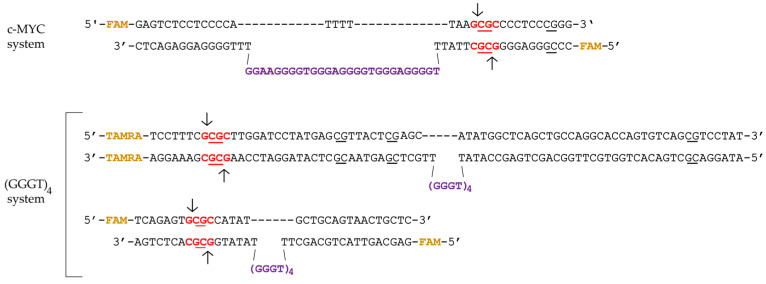
DNA models: *c-MYC* system (containing the *c-MYC* promoter sequence) and (GGGT)_4_ system. CpG sites are underlined; TAMRA and FAM (in yellow) indicate fluorophores; the R.Hin6I site is in red and arrows indicate cleavage site; G4 motifs are highlighted in purple.

In this study, we developed a set of synthetic DNA models harboring G-rich sequences derived from the promoter region of the *c-MYC* oncogene (*c-MYC* G4). This set (the c-MYC system in Table 1, Figure 1) includes (i) a 27-nt FAM-labeled oligonucleotide (27G4) and its mutant form (27G4-mut) containing two G > A substitutions to prevent quadruplex formation for control experiments [27]; (ii) a perfect 58-bp DNA duplex (c-MYC_58Uf/58Df) and a duplex carrying an extrahelical insert of 27-nt in one of the strands, capable of folding into a G4 structure (c-MYC_35Uf/62Df_G4) (Table 1, Figure 1). Oligonucleotides of arbitrary sequences (30X) and a known 30-bp MTase substrate (30Uf/30Df) were used as controls.

In addition, fluorescently labeled DNA duplexes were constructed with an extrahelical G4 motif (GGGT)_4_ located at varying distances from the CpG site to be methylated: proximal (32Uf/51Df_G4) and distal (76Uf/95Df_G4) (system (GGGG)_4_ in Table 1 and Figure 1). The (GGGT)_4_ motif, commonly found in eukaryotic genomes, folds into an exceptionally stable three-tetrad parallel G4 [24]; the duplex flank sequences in 32Uf/51Df_G4 and 76Uf/95Df_G4 are arbitrary. For comparison, we used the perfect DNA duplexes 32Uf/32Df and 76Uf/76Df, which cannot form G4 structures (Table 1). Moreover, the same DNA molecules without fluorescent labels were generated for both systems.

To create DNA duplexes with stable G4 structures, we employed an approach that prevented the formation of a competing Watson-Crick double helix [28]. These DNA models were obtained via hybridizing partially complementary strands, one of which contained a G4 motif flanked by oligonucleotide fragments, while the opposite strand lacked a site complementary to the quadruplex-forming sequence.

A significant advantage of engineered DNA duplexes containing an extrahelical G4 structure is the ability to examine the methylation of specific CpG located within the restriction endonuclease R.Hin6I recognition site (see below) in the duplex flanks, and to analyze the G4 impact on the methylation level directly within one duplex system.

### 2.2. Formation, Topology, and Thermal Stability of c-MYC G4 in the Designed DNA Models

We applied a combination of UV spectroscopy and circular dichroism (CD) to characterize the developed models. CD makes it possible to determine the G4 topology, whereas UV spectroscopy allows for independent monitoring of the G4 and DNA duplex unfolding and estimation of their melting temperature (T_m_). Temperature-dependent UV absorption at 295 nm is known to be a marker of the G4 structure [29]. Unlike the DNA duplex, whose melting is accompanied by a hyperchromic effect (usually at 260 nm), the melting of the G4 at 295 nm causes a decrease in optical density. Using CD spectroscopy, the T_m_ values of the DNA secondary structures were determined independently. It is important to note that oligonucleotides without fluorescent labels were used for these physicochemical studies. The measurements were carried out in buffer solutions containing either 100 mM KCl, which corresponds to the conditions of Dnmt3a functioning (buffer A), or 2.5 mM KCl (to capture the entire conformational transition region within the available temperature range and compare the T_m_ values of G4 structures in different DNA models) (buffer B).

**Figure 2:**
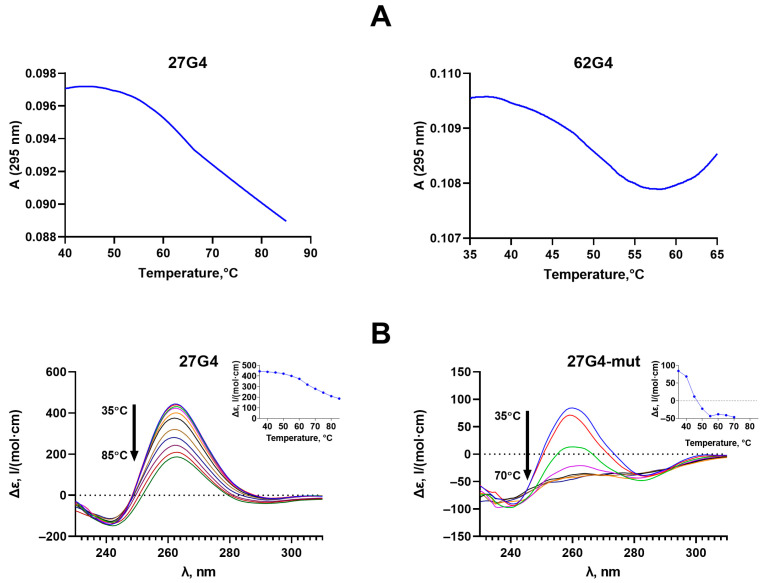
Thermal stability and topology of G4 structures formed by oligonucleotides 27G4, 27G4-mut, and 62G4. Temperature-dependent UV absorption measured at 295 nm (~3 µM oligonucleotide strand concentration) in buffer B (**A**). CD spectra recorded at increasing temperatures, starting from 35 °C (upper curve) with a step of 5 °C, are shown using multicolored lines. (Insets) CD-monitored melting profiles at the wavelength corresponding to the positive maximum of the CD spectrum (~2 µM oligonucleotide strand concentration); 27G4 was measured in buffer B, 27G4-mut–in buffer A (**B**).

The UV melting profiles of oligonucleotides 27G4 and 62G4, differing in the length of the nucleotide sequences flanking the G4 motif, showed one-step conformational transitions corresponding to the G4 unfolding, with an observed hypochromic effect and T_m_ values of 66 and 46 ± 1 °C, respectively (Figure 2A). The decrease in T_m_ value for 62G4 compared to 27G4 is explained by an unfavorable entropy effect due to the significant elongation of the G4 flanks. The same factor is responsible for reducing the hypochromic effect accompanying G4 unfolding in 62G4 compared to 27G4; an additional 35 nucleotide residues in 62G4 absorb UV light but do not contribute to the G4 unfolding.

**Figure 3:**
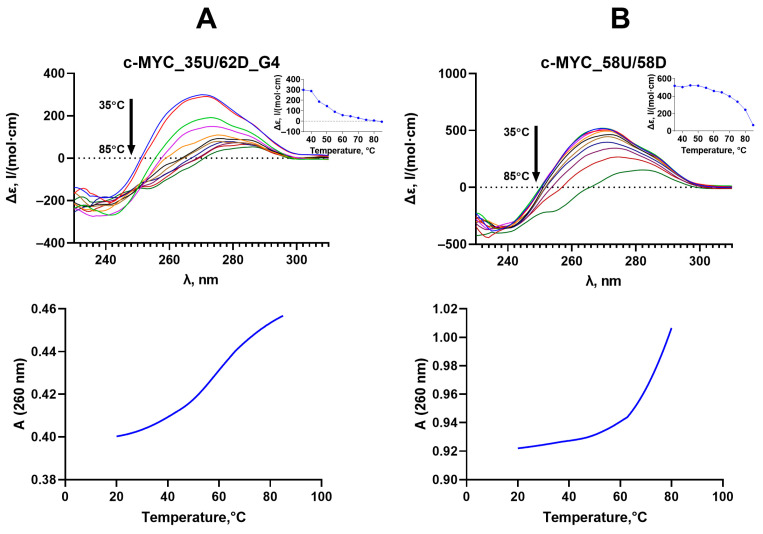
Thermodynamic stability and topology of c-MYC_35U/62D_G4 containing the *c-MYC* G4 structure stabilized in a duplex surrounding (**A**) and a control perfect duplex c-MYC_58U/58D derived from the *c-MYC* promoter region (**B**). Upper panel: CD spectra recorded at increasing temperatures, starting from 35 °C (upper curve) with a step of 5 °C, are shown using multicolored lines. (Insets) CD-monitored melting profiles at the wavelength corresponding to the positive maximum of the CD spectrum (~2 µM oligonucleotide strand concentration). Lower panel: temperature dependence of UV absorption at 260 nm. All measurements were carried out in buffer B.

These results were supported by CD data (Figure 3B). The CD spectrum of 27G4 at 35 °C revealed a typical parallel G4 fold with positive peak at 264 nm and a negative one at 245 nm. CD spectra recorded at different temperatures made it possible to obtain melting profiles for the DNA models studied (Figure 2B, insets). As can be seen, the T_m_ of 65 ± 1 °C derived from the CD data for 27G4 is in good agreement with the T_m_ value obtained from the temperature dependence of UV absorbance at 295 nm (66 ± 1 °C). Given that both methods used are highly correlated, we took advantage of each approach to characterize the topology and/or the thermodynamic stability of G4 structures without duplicating the results of both methods. Thus, the CD data showed that 27G4-mut, containing two G > A substitutions, still retains the G4 structure with a T_m_ value of 43 ± 1 °C (Figure 2B); these measurements were performed in buffer A containing 100 mM KCl and used for the MTase-mediated methylation assay, but not in buffer B with 2.5 mM KCl, which is typically applied for our spectroscopic studies.

The parallel *c-MYC* G4 structure with T_m_ = 51 ± 1 °C is maintained when the quadruplex motif is flanked by DNA duplexes (Figure 3A, upper panel). According to CD data, the positive band of c-MYC_35U/62D_G4 is shifted to longer wavelengths due to the contribution of duplex flanks. While the G4 domain of c-MYC_35U/62D_G4 was monitored by CD spectroscopy, the thermal stability of duplex regions in the same DNA model was tested using UV melting (T_m_ = 65 ± 1 °C) (Figure 3A, lower panel). The observed melting profile at 260 nm demonstrates a hyperchromic effect and reflects the superposition of the melting of two duplex regions flanking the G4 insert (T_m_ = 65 ± 1 °C).

The addition of a fully complementary oligonucleotide to a DNA strand containing the G4 motif is known to shift the conformational equilibrium toward the B-form double helix [30]; in this case, the G4 formation becomes thermodynamically unfavorable. Using a combination of CD and UV spectroscopy, it was shown that hybridization of two fully complementary 58-nt DNA strands results in the formation of an extremely stable DNA duplex (c-MYC_58U/58D), and even minor G4 folding is not observed (Figure 3B).

The secondary structure of the engineered (GGGT)_4_-containing system used for comparative experiments (Table 1), as well as the G4-folding topology and the thermal stability of both the G4 and duplex domains, has been previously analyzed using various biophysical and biochemical techniques [28].

### 2.3. Dnmt3a-CD Effectively Binds to Oligonucleotide 27fG4

Dnmt3a consists of a C-terminal catalytic domain (Dnmt3a-CD) and an N-terminal regulatory region responsible for targeting chromatin and interactions with other proteins [7,8]. The regulatory region includes the PWWP and ADD domains. Dnmt3a-CD possesses catalytic activity in the absence of the regulatory region. The active form of Dnmt3a is a tetramer with two active sites, which, in complex with DNA, spans one turn of the double helix [31]. It is assumed that the tetrameric form of Dnmt3a-CD can further oligomerize on DNA [32]. The amino acid sequence of murine Dnmt3a-CD is 98% identical to that of human DNMT3A-CD [33]. The prokaryotic CpG-recognizing monomeric MTase M.SssI was also used as a control; the amino acid sequence of M.SssI contains all ten conserved motifs typical of C5-MTases, directly responsible for the catalytic reaction [34,35].

**Figure 4:**
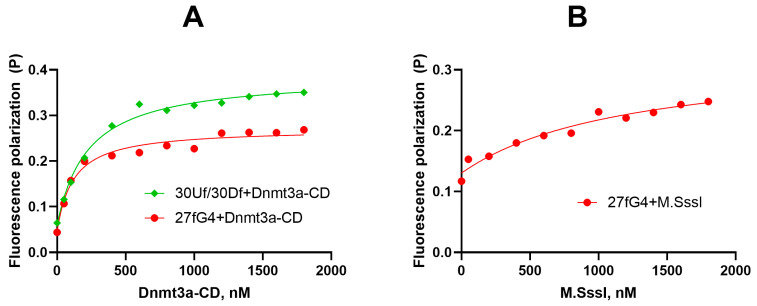
Binding curves of FAM-tagged 27fG4 and 30Uf/30Df to Dnmt3a-CD (**A**) and M.SssI (**B**) derived from fluorescence polarization data. A amount of 10 nM DNA, 100 µM AdoHcy, 20 mM HEPES-NaOH (pH 7.5), 100 mM KCl, 1 mM EDTA, 1 mM 1,4-dithiothreitol, 0–1800 nM Dnmt3a-CD or M.SssI.

Here, we examined the binding of Dnmt3a-CD and M.SssI to the G4 structure formed by the purine-rich strand of the promoter region of the cancer-associated *c-MYC* gene (27fG4). Fluorescence polarization was used to quantitatively characterize the parameters of Mtases-G4 complexation in solution. We performed direct titration of the fluorescently labeled oligonucleotide 27fG4 and the control perfect DNA duplex 30Uf/30Df with Dnmt3a-CD or M.SssI in the presence of S-adenosyl-L-homocysteine (AdoHcy), an analog of the S-adenosyl-L-methionine (AdoMet) cofactor that facilitates the formation of a specific complex between MTase and DNA. The enzyme–DNA binding curves presented in Figure 4 suggest that the G4-containing oligonucleotide 27fG4 forms a strong complex with Dnmt3a-CD with an equilibrium dissociation constant, *K*_d_, (120 ± 30 nM), which is approximately two times less than the *K*_d_ value (220 ± 30 nM) for the double-stranded substrate 30Uf/30Df lacking the G4 structure. In comparison, the PWWP regulatory domain of Dnmt3a binds to 27fG4 less efficiently than the CD domain (*K*_d_ value 0.9 ± 0.2 µM) (Figure S1).

Notably, M.SssI binds to 27fG4 quite poorly (Figure 4B); the *K*_d_ value calculated for this complex (1.2 ± 0.6 µM) was 10 times higher than for Dnmt3a-CD•27fG4•AdoHcy. The same patterns were found for the M.SssI complex with the 19-nt 5′-TT(GGGT)_4_TT, forming a parallel G4 structure [36]. 

### 2.4. Inhibition of a 30-bp DNA Duplex Methylation in the Presence of 27G4

**Figure 5:**
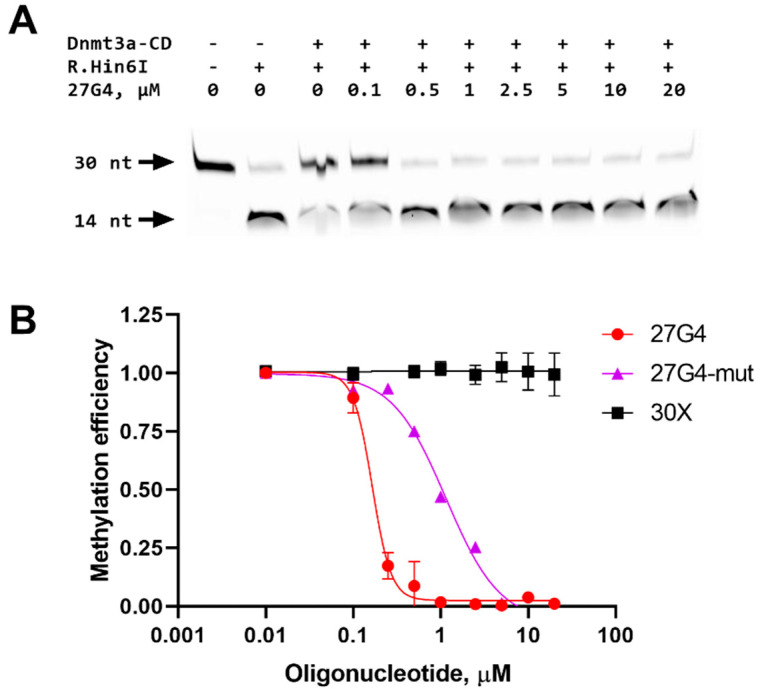
Inhibition of Dnmt3a-CD-mediated methylation of DNA duplex 30Uf/30Df (300 nM) by the G4-forming oligonucleotide 27G4. The products of 30Uf/30Df cleavage induced by the R.Hin6I after its methylation with 2 µM Dnmt3a-CD in buffer A in the presence of 25 µM AdoMet and 27G4 (27G4 concentrations indicated above the gel lanes) were analyzed on 20% polyacrylamide gel containing 7 M urea (**A**). Methylation efficiency is plotted against the concentration of 27G4 or controls: 30X lacking G4 and 27G4-mut. Error bars represent SEM from at least three independent experiments (**B**).

To determine whether G-quadruplexes could inhibit DNA methylation, the impact of 27G4 on the methylation of 30Uf/30Df was studied. The extent of methylation by Dnmt3a-CD was measured using a procedure similar to that described previously [37]. Specifically, 30Uf/30Df was methylated with Dnmt3a-CD followed by treatment with the endonuclease R.Hin6I, which cleaves only the unmethylated GCGC site overlapping the CpG site. Cleavage products were visualized in a denaturing polyacrylamide gel (Figure 5A), and the extent of methylation was calculated as the ratio of the fluorescence intensity of the 14-nt cleavage product to the total fluorescence intensity of the intact and cleaved DNA. 

In the absence of 27G4, the 30Uf/30Df duplex was almost completely methylated (Figure 5B). With increasing 27G4 concentration, the extent of 30Uf/30Df methylation decreased, indicating the inhibition of Dnmt3a-CD activity. The IC_50_ value determined from a plot of percent methylation, R, versus 27G4 concentration (Figure 5B) was 160 ± 10 nM. In contrast, the presence of the non-G4 control oligonucleotide 30X did not result in the inhibition of enzyme activity. The mutated control 27G4-mut turned out to be a significantly weaker MTase inhibitor (IC_50_ = 1100 ± 200 nM) compared to 27G4, which is consistent with CD data on the extremely low stability of the G4 structure formed by this oligonucleotide. Thus, 27G4 binds tightly to Dnmt3a-CD, resulting in the inhibition of Dnmt3a-CD methylation activity by preventing MTase from binding to its regular DNA substrate.

### 2.5. Effect of c-MYC G4 on Methylation of the c-MYC Promoter Region

**Figure 6:**
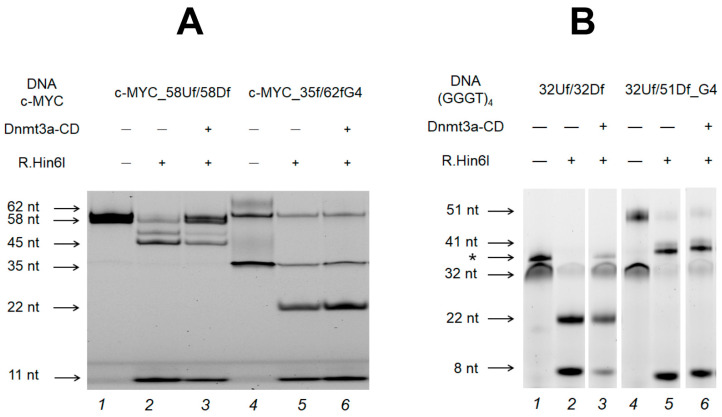
Effect of the G4 structure in c-MYC_35Uf/62Df_G4 (**A**) and in (GGGT)_4_-containing 32Uf/51Df_G4 (**B**) on their methylation with Dnmt3a-CD in comparison with the control c-MYC_58Uf/58Df (**A**) and 32Uf/32Df (**B**). The products of DNA duplex (75 nM) cleavage by R.Hin6I after methylation with Dnmt3a-CD (2.5 µM) were separated on a 10% polyacrylamide gel containing 7 M urea. DNA duplexes that do not dissociate during gel electrophoresis are marked with an asterisk.

**Figure 7:**
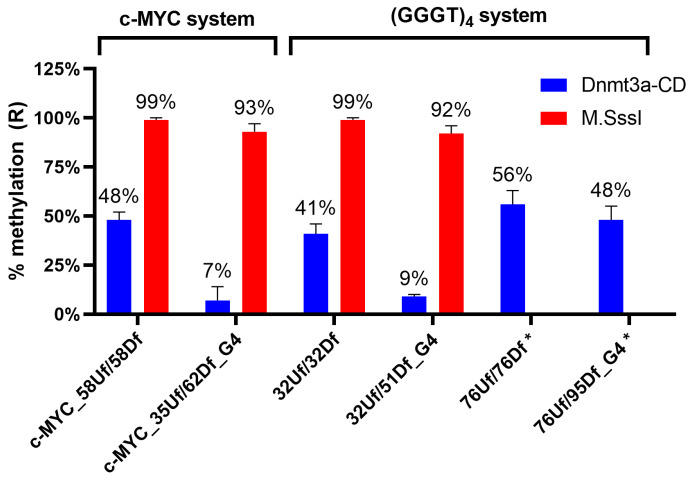
Extent of methylation of model DNA duplexes (75 nM) with Dnmt3a-CD (2.5 µM) and M.SssI (1 µM). Error bars represent the SDs derived from at least three independent experiments. * Adapted from [36].

Further, we analyzed the impact of the G4 formed and stabilized within the double-stranded *c-MYC* promoter region (c-MYC_35Uf/62Df_G4) on the methylation activity of Dnmt3a-CD. For comparison, G4-free c-MYC_58Uf/58Df, which mimics the native region of the *c-MYC* promotor, was used (Table 1, Figure 1). The methylation extent of c-MYC_58Uf/58Df and c-MYC_35Uf/62Df_G4 were determined as described above. FAM-labeled DNA cleavage products were analyzed using denaturing polyacrylamide gel (Figure 6A), followed by calculating the extent of methylation (Figure 7).

Figure 6 shows that after the treatment of the perfect c-MYC_58Uf/58Df duplex with Dnmt3a-CD (lane 3), the intensity of the bands corresponding to the 11 and 45 nt cleavage products decreased compared to that of the c-MYC_58Uf/58Df not treated with Dnmt3a-CD (lane 2). These data indicate that a significant portion of this duplex underwent methylation. On the contrary, the quantity of 11 nt and 22 nt cleavage products of the *c-MYC* G4-containing c-MYC_35Uf/62Df_G4 substrate before and after methylation (lanes 5 and 6) remained almost identical, indicating the low efficiency of methylation of a DNA duplex with an embedded extrahelical G4. Overall, the formation of a G4 in the c-MYC_35Uf/62Df_G4 simulating a fragment of the *c-MYC* oncogene promoter leads to a seven-fold decrease in methylation efficiency compared to the c-MYC_58Uf/58Df DNA duplex, which is not capable of forming a G4 (Figure 7).

### 2.6. Influence of the Relative Positions of G4 and CpG Sites within a DNA Duplex on the Methylation Activity of Dnmt3a-CD

To evaluate the role of the distance between the G4 and the analyzed CpG site on the functioning of Dnmt3a-CD, two DNA duplexes with an embedded stable parallel G4 structure formed by (GGGT)_4_ folding were compared. These included the new model duplex 32Uf/51Df_G4 and the longer, previously studied 76Uf/95Df_G4 [36], which differed in the distance between the CpG site and the G4 structure: 6 nt and 28 nt, respectively (Table 1 and Figure 1). In both cases, the efficiency of methylation of CpG located within the R.Hin6I site was examined and compared with the efficiency of the corresponding control DNA duplexes lacking G4 structures. Figure 6B shows the cleavage product bands (8 nt and 41 nt) for 32Uf/51Df_G4, as well as for the control 32Uf/32Df before and after methylation. As can be seen, the G4 formation in close proximity (6 nt) to the CpG site reduces the methylation efficiency by approximately five times compared to that for a perfect DNA duplex (Figure 6B and Figure 7). In contrast, the hypomethylation effect induced by distal G4 (28 bp) is negligible (Figure 7). Thus, moving the methylation site away from the G4 significantly reduces the impact of this non-B-DNA structure on Dnmt3a-CD activity.

### 2.7. M.SssI-Mediated Methylation of Model DNA with G4 Structure

**Figure 8:**
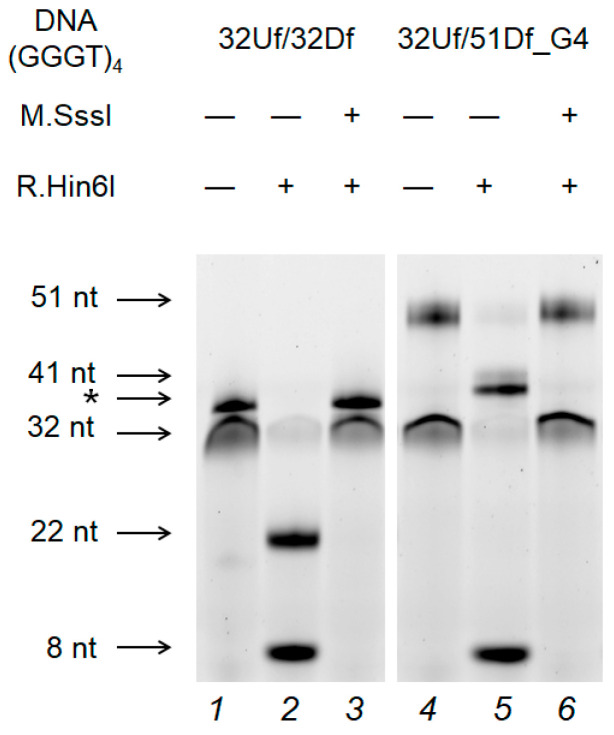
Effect of G4 formation in 32Uf/51Df_G4 on M.SssI-mediated methylation compared to the control 32Uf/32Df. The cleavage products of the DNA duplex (75 nM) by R.Hin6I after methylation with Dnmt3a-CD (1.0 µM) were separated on a 10% polyacrylamide gel containing 7 M urea. DNA duplexes that do not dissociate during gel electrophoresis are marked with an asterisk.

To address the mechanism of the G4 effect on CpG methylation, experiments were carried out using the prokaryotic CpG-recognizing MTase M.SssI and a DNA duplex (32Uf/51Df_G4) containing extrahelical stable G4 as a substrate. It was found that the quadruplex structure does not affect the efficiency of M.SssI-induced methylation (compare the methylation level in 32Uf/51Df_G4 and 32Uf/32Df lacking G4 in Figure 7 and Figure 8, and in a pair of substrates belonging to the c-MYC system: c-MYC_35Uf/62Df_G4 and c-MYC_58Uf/58Df (Figure 7)). 

As indicated in Figure 7, even when a G4 is formed in close proximity to the CpG site, as in 32Uf/51Df_G4 and c-MYC_35Uf/62Df_G4, the activity of M.SssI is not affected, in contrast to Dnmt3a-CD, for which G4 formation becomes an obstacle to normal functioning.

## 3. Discussion

Both DNA methylation and G4 structures play multiple roles in cancer biology, DNA replication, and gene regulation. Therefore, the experimental evidence for crosstalk between *c-MYC* G4s and the functioning of the eukaryotic Dnmt3a presented in our in vitro study is of great importance. To address this issue, we used the catalytic domain of Dnmt3a and designed DNA models containing *c-MYC* G4: a single-stranded 27-nt oligonucleotide 27G4 mimicking the G4 motif (Pu27) in the *c-MYC* promoter, and a DNA duplex (c-MYC_35Uf/62Df_G4) bearing an extrahelical Pu27 insert in the center of one of its strands (Figure 1).

Using CD and UV spectroscopy, we revealed that 27G4 forms a stable *c-MYC* G4 structure with a parallel topology under Dnmt3a-CD operating conditions. The parallel topology and high thermal stability of the G4 structure were maintained when the 27G4 sequence was embedded into a double-stranded *c-MYC* promoter sequence lacking the fragment complementary to 27G4 (c-MYC_35Uf/62Df_G4) (Figure 3B). This approach, allowing for stabilization of the G4 structure in a duplex context, was elaborated to prevent its conformational transition to the more energetically favored B-DNA [28] (Figure 3A). The native *c-MYC* promoter fragment (c-MYC_58Uf/58Df) adopts a B-form DNA structure under Dnmt3a-CD operating conditions.

We further established that Dnmt3a-CD forms a stable-specific complex with 27G4f. Comparison of the Dnmt3a-CD binding affinity to this oligonucleotide and to a known 30-bp DNA substrate, 30Uf/30Df, revealed a marked preference for the parallel G4 structure formed by 27G4f. This result is in line with the high binding affinity of full-length mammalian MTases and other proteins to G4 structures [23,24,38]. Specifically, these MTases (human DNMT3A, DNMT3B, and DNMT1) show a higher binding preference to a G4-forming oligonucleotide mimicking the G4 motif in the *c-MYC* promoter compared to the control non-G4 oligonucleotides [23,24]. Notably, these MTases share a conserved catalytic C-terminal domain with considerable sequence similarity, especially within the DNMT3 family [7]. According to our previous data [36] and the results of the present study, Dnmt3a-CD forms strong complexes with two types of parallel G4 structures: (GGGT)_4_ and *c-MYC* G4, which differ mainly in sequence composition and length of the loops. These findings imply that Dnmt3a-CD efficiently recognizes the parallel quadruplex scaffold but does not discriminate between G4 loop isomers.

27G4 was shown to promote significant inhibition of Dnmt3a-CD activity, in contrast to the control oligonucleotide of random sequence, 30X (Figure 5B); these data confirm the binding specificity of Dnmt3a-CD to the quadruplex structure formed in 27G4. The mutant oligonucleotide 27G4-mut was shown to be a weak inhibitor of Dnmt3a-CD activity due to low G4 stability (Figure 2B). Similar methylation inhibition effects induced by the *c-MYC* G4 oligonucleotide were found for the human maintenance MTase DNMT1 [24]. The IC_50_ value for 27G4 is comparable to the IC_50_ values of known Dnmt3a-CD inhibitors, such as olivomycin A, which binds to the DNA minor groove [39], and the DNA-intercalating curaxin CBL0137 [37]. 

Inhibition of Dnmt3a-CD induced by 27G4 suggests the hypomethylation of the *c-MYC* oncogene promoter due to *c-MYC* G4 binding to the enzyme. However, single-stranded oligonucleotides folded into G4 structures are not informative when studying the role of G4 in regions flanking CpG sites in the methylation process. Much more suitable are the combined duplex–quadruplex models, containing a *c-MYC* G4 structure stabilized in the DNA duplex context (e.g., c-MYC_35Uf/62Df_G4). In addition, the created model made it possible to monitor the methylation of specific CpG sites. It was previously shown that G4 formation in long dsDNA is promoted by conditions of molecular crowding created by PEG [40]. However, the G4 stabilization observed under these conditions occurs only during the process of in vitro transcription or heat denaturation/renaturation and is sequence-dependent. 

In the DNA duplex c-MYC_35Uf/62Df_G4, the CpG site, which is the methylation target, is located at a distance of 6 bp from G4 (Table 1). It was shown that the degree of-MYC_35Uf/62Df_G4 methylation, decreased by approximately seven times compared to the standard perfect DNA duplex c-MYC_58Uf/58Df (Figure 7). This is the first direct evidence that parallel G4 down-regulates Dnmt3a activity. 

To answer the question of whether the G4 effect on the methylation level persists when the CpG is moved away from the quadruplex structure, we compared the degrees of methylation of 32Uf/51Df_G4 and 76Uf/95Df_G4, containing an embedded extrahelical (GGGT)_4_ motif, which differed in the distance between the G4 and the analyzed CpG site in flanking duplexes (Figure 7). Removal from the G4 structure on 28 bp from the analyzed CpG site (76Uf/95Df_G4) significantly reduces the hypomethylation effect of the G4. DNA duplexes 32Uf/51Df_G4 and c-MYC_35Uf/62Df_G4 with parallel G4 insertions within the duplex structure located at approximately the same short distance from the CpG site showed an equally high reduction in methylation compared to DNA substrates lacking G4.

Thus, one would expect DNA hypomethylation near the *c-MYC* G4 structure and maintenance of elevated methylation levels in oncogene promoter regions distal to the G4. Previous studies examined the impact of the relative position of quadruplex structures and CpG sites in gene promoters on the methylation status of CpG islands based on whole-genome bisulfite sequencing data [24,41]. It was noted that the hypomethylating effect of the G4 was inversely proportional to the distance between the G4 and the CpG, but this analysis did not take into account the activity of DNA methyltransferases [41].

Understanding the molecular-level interactions between G4 structures in the *c-MYC* promoter region and Dnmt3a-CD remains unclear. High-resolution structural information on the nature of G4 interactions with various cell proteins and their selectivity in recognizing certain G4 structures is extremely limited [5]. Structural studies of Dnmt3a complexes with G4 DNA have not been performed. Two main mechanisms by which various proteins interact and function with intracellular G4s have been discussed [14,42]: (i) G4-unfolding proteins can unfold the G4 structure after binding to it, acting as helicases, and (ii) G4-recruited proteins can bind to G4 structures in specific functional regions of the DNA. We hypothesize that G4 structures may also serve as an obstacle to the proper formation of the DNA–protein interface. Obviously, the hypomethylation effect that we discovered in vitro for Dnmt3a-CD is primarily due to the effective binding of MTase to the non-canonical structure in c-MYC_35Uf/62Df_G4. However, it is also necessary to take into account the ability of G4 to sterically interfere with the Dnmt3a-mediated methylation of nearby CpG sites, preventing the formation of the correct DNA–protein complex. These assumptions are supported by the clear dependence of methylation efficiency on the distance between the G4 and the CpG (Figure 7) and by comparative experiments with the prokaryotic MTase M.SssI. 

Despite the substantial similarity of the primary structures of M.SssI and Dnmt3a-CD, namely the presence of ten conserved amino acid motifs responsible for the catalytic reaction [7,35], profound differences were revealed in their interaction with G4. In the case of M.SssI, the G4 impact on the methylation of duplex–quadruplex models was negligible (Figure 7). Furthermore, M.SssI exhibits much weaker binding to 27G4f (Figure 4B) or TT(GGGT)_4_TT oligonucleotide [36] compared to Dnmt3a-CD. The key difference between these two enzymes may be the variable structure and composition of their complexes with DNA substrate [31,43]: firstly, the oligomerization of Dnmt3a-CD leads to the inability to properly bind to the double-stranded region of our model c-MYC_35Uf/62Df_G4, in contrast to the standard binding of monomeric M.SssI; secondly, the specific structure of the DNA-binding cavity in Dnmt3a formed by four monomers of the enzyme. Overall, the ability to tetramerize is likely a key characteristic that determines the Dnmt3a-CD’s affinity for parallel G4 structures. Dnmt3a-CD tetramerization results in the development of a DNA binding surface that appears to be more specific for G-quadruplex structures than that of the monomeric M.SssI.

## 4. Materials and Methods

### 4.1. Reagents

All oligonucleotides (Table 1) were commercial products (Eurogene, Moscow, Russia). Some of the oligonucleotides contained the fluorescent dye 6-carboxyfluorescein (FAM) or tetramethylrhodamine (TAMRA). The concentrations of oligonucleotides were determined spectrophotometrically, as described previously [28]. AdoMet and AdoHcy (Sigma, Steinheim, Germany) were used in the study. The following buffer solutions were used: A—20 mM HEPES-NaOH (pH 7.5), 100 mM KCl, 1 mM EDTA, 1 mM 1,4-dithiothreitol; B—10 mM Tris-HCl (pH 7.3), 70 mM NaCl, 2.5 mM KCl. G4 structures in oligonucleotides and DNA duplexes were formed using annealing (by heating at 95 °C for 3 min and slowly cooling to 4 °C) in an appropriate buffer; DNA duplexes, including those containing G4 structures, were prepared by annealing complementary (or partly complementary) DNA strands under the same conditions.

### 4.2. Enzymes

To obtain Dnmt3a-CD, *Escherichia coli* BL21(DE3) cells were transformed with plasmid pET-28a(+) carrying the gene encoding Dnmt3a-CD with an N-terminal 6 × His tag. Subsequently, Dnmt3a-CD was isolated and purified using metal-affinity chromatography on Co^2+^-containing TALON^®^ resin (GE Healthcare, Chicago, IL, USA) [43]. M.SssI and PWWP domain of Dnmt3a were purified as previously described [34] and [36], respectively. Plasmids pET-28a(+) encoding Dnmt3a-CD and pGEX-6P-2 encoding the PWWP domain were provided by Prof. A. Jeltsch (University of Stuttgart, Stuttgart, Germany). R.Hin6I is a commercial product (SibEnzyme, Nowosibirsk, Russia). The purity of the protein samples was evaluated using electrophoresis in a 12% SDS-polyacrylamide gel. The protein concentrations were determined using the Bradford assay per protein monomer. Protein preparations were stored at −80 °C.

### 4.3. CD Measurements

CD spectra of oligonucleotides and DNA duplexes that do not contain a fluorescent label were recorded in a quartz cuvette of 10 mm optical path length at room temperature or between 35 to 80 °C with a step of ~5 °C at an average heating rate of 0.5 °C/min in buffer B or A on a Chirascan CD spectrometer (Applied Photophysics Ltd., Surrey, UK) equipped with a thermoelectric controller. The DNA concentration (~2 µM concentration per oligonucleotide strand) was chosen to attain an absorption of 0.6–0.8 at 260 nm, which gives an optimum signal-to-noise ratio. The measurements were performed in the 230–320 nm wavelength range at a scanning speed of 30 nm/min and a signal averaging time of 2 s with a constant flow of dry nitrogen. The CD spectra were baseline-corrected for signal contributions caused by the buffer and processed with Graph Pad Prism 8.0.1 (Graph Pad Software, San Diego, CA, USA). CD spectra were plotted as molar dichroism per oligonucleotide strand against wavelength. The CD melting profiles revealed the temperature dependence of a CD signal at a specific wavelength.

### 4.4. UV Spectroscopy Melting of Oligonucleotides Containing G4 Motifs and DNA Duplexes

UV absorbance versus temperature profiles of DNA samples (at ~3 µM concentration per oligonucleotide strand) were recorded in a 600-μL quartz microcuvette with an optical path length of 10 mm on a double-beam U-2900 UV/visible spectrophotometer (Hitachi, Tokyo, Japan) equipped with a Hitachi thermoelectric controller. UV absorbance changes were monitored between 20 and 85 °C at 295 or 260 nm at a heating rate of 1.0 °C/min. T_m_ value, defined as the temperature of the mid-point, was estimated from a maximum/minimum value of the first derivative of the fitted curve for data smoothed with the Savitzky–Golay filter.

### 4.5. Complex Formation of Dnmt3a-CD and M.SssI with DNA

Complex formation was investigated using a fluorescence polarization assay via direct titration of FAM-labeled oligonucleotides or DNA duplexes with Dnmt3a-CD, M.SssI or PWWP in the presence of AdoHcy as described before [37]. Various amounts of the protein were added to a mixture containing 10 nM DNA substrate in buffer A with 100 µM AdoHcy, and fluorescence polarization (*P*) was measured using a Cary Eclipse spectrofluorometer (Varian). The *P* value was calculated according to the equation *P* = (*I*_v_ − *G*·*I*_h_)/(*I*_v_ + *I*_h_), where *I*_v_ and *I*_h_ are vertical and horizontal components of emitted light, respectively, and *G* is a correction factor. The experimental data are presented as a dependence of *P* on the total concentration of the MTase. The titration curves of each DNA duplex with the MTase were obtained in at least two technical replicates. Dissociation constants (*K*_d_) of the complexes were obtained by fitting the observed *P* values to the single-site binding equation in Graph Pad Prism 8.0.1 (Graph Pad Software, San Diego, CA):

$$P=P_{0}+\frac{P_{\max}\cdot C_{\mathrm{enz}}}{K_{d}+C_{\mathrm{enz}}}$$

where *P* is fluorescence polarization, *P*_0_ is initial fluorescence polarization of unbound DNA, *P*_max_ is fluorescence polarization at maximum binding, *C*_enz_ is enzyme concentration, and *K*_d_ is equilibrium dissociation constant.

### 4.6. Methylation of DNA Duplexes

Methylation activity of Dnmt3a-CD and M.SssI was analyzed with the protection of methylated DNA from cleavage by R.Hin6I (G↓CGC) [43]. FAM- or TAMRA-labeled DNA duplexes containing or not containing G4 structures (75 nM) were incubated in buffer A for 2 h at 37 °C in the presence of Dnmt3a-CD (2.5 µM) or M.SssI (1 µM) and 25 µM AdoMet. After methylation, the samples were treated with proteinase K (100 µg/mL) for 30 min at 55 °C to disrupt the Dnmt3a-CD•DNA complex and facilitate the entry of the DNA strands into a polyacrylamide gel. Proteinase K was subsequently inactivated via heating of the samples to 95 °C followed by slow cooling to room temperature. Next, the mixtures were incubated with 1 U of R.Hin6I for 1 h at 37 °C in the presence of 3 mM Mg^2+^. In control mixtures, cleavage was performed without prior methylation. The mixtures were analyzed using electrophoresis in a 10% polyacrylamide gel with 7 M urea. The resulting gels were visualized using a Typhoon FLA 9500 scanner (GE Healthcare Life Sciences, Buckinghamshire, UK), and the fluorescence intensities of intact DNA and cleavage products were determined. The extent of DNA cleavage (*w*) was calculated using GelQuantNET version 1.7.8. The extent of methylation (*R*) was calculated using the equation:

$$R=\frac{w_{0}-w_{\mathrm{Dnmt}3a}}{w_{0}},$$

where *w*_0_ is the extent of DNA cleavage before methylation, *w*_Dnmt3a_ is the extent of DNA cleavage after methylation by Dnmt3a-CD.

When calculating the extent of cleavage, we considered the total fluorescence intensities of the initial DNA duplexes, because under denaturing conditions, the strands do not separate completely due to the high stability of the extended DNA double helix. The fluorescence intensities of the cleavage products were also summed in the calculations.

### 4.7. Inhibition of Methylation of a Standard DNA Substrate by a G4 Oligonucleotide

A FAM-labeled DNA duplex (30Uf/30Df) containing a CpG site within the R.Hin6I site (300 nM) was incubated in buffer A for 1 h at room temperature at various concentrations of the 27G4 oligonucleotide. 27G4-mut and 30X oligonucleotides were used as controls. Subsequently, the 30Uf/30Df was methylated by 2 µM Dnmt3a-CD in the presence of 25 µM AdoMet for 1.5 h at 37 °C [36]. Then, R.Hin6I cleavage was performed, and the mixtures were analyzed on 20% polyacrylamide gels containing 7 M urea, with the determination of the extent of methylation as described above.

IC_50_ values were calculated via fitting the dependence of the extent of methylation on the concentration of 27G4 using Graph Pad Prism 8.0.1 (Graph Pad Software, San Diego, CA, USA).

## 5. Conclusions

The *c-MYC* oncogene, overexpressed in the majority of solid tumors, is a well-known gene with a G4-forming sequence in its promoter region.

In this study, we demonstrated the strong specific binding of Dnmt3a-CD to the oligonucleotide 27G4 folded into a parallel *c-MYC* G4 structure. The *c-MYC* G4–enzyme complex inhibits Dnmt3a-CD activity by preventing standard DNA substrate binding to the enzyme. Using a specially designed DNA construct that mimics the G/C-rich promoter region of the *c-MYC* oncogene and stabilizes *c-MYC* G4 in a duplex context, we found that the presence of *c-MYC* G4 reduces the methylation activity of Dnmt3a-CD. The hypomethylation effect depends on the distance between the G4 and the methylated CpG site. It can be assumed that the G4 effect on the Dnmt3a-CD function is determined by the sequestration of the enzyme on this non-canonical structure and the disruption of the enzyme’s proper binding to the double-stranded region of the *c-MYC* promoter. In vivo, G4 formation in the *c-MYC* oncogene promoter may be one of the reasons for DNA hypomethylation resulting in the overexpression of the c-MYC protein and cancer progression. Overall, G4 formation in gene promoters partially explains their hypomethylation compared to the rest of the genome [24,41]. 

Our findings and hypotheses contribute to the understanding of the relationships between the promoter G4’s ability to bind MTases, DNA methylation activity, and functional consequences. Future research should provide clear evidences of the in vivo effect of G4 structures on the methylation machinery. It is known that genome G4 structures may interfere with the fidelity of DNA replication and repair. Their involvement in the regulation of DNA methylation revealed in this study suggests a complex network of G4–protein interactions that govern the main biological processes associated with genome stability and oncogenesis.

## Figures and Tables

**Table 1:** Designation and sequence of oligonucleotides and DNA duplexes used in this work.

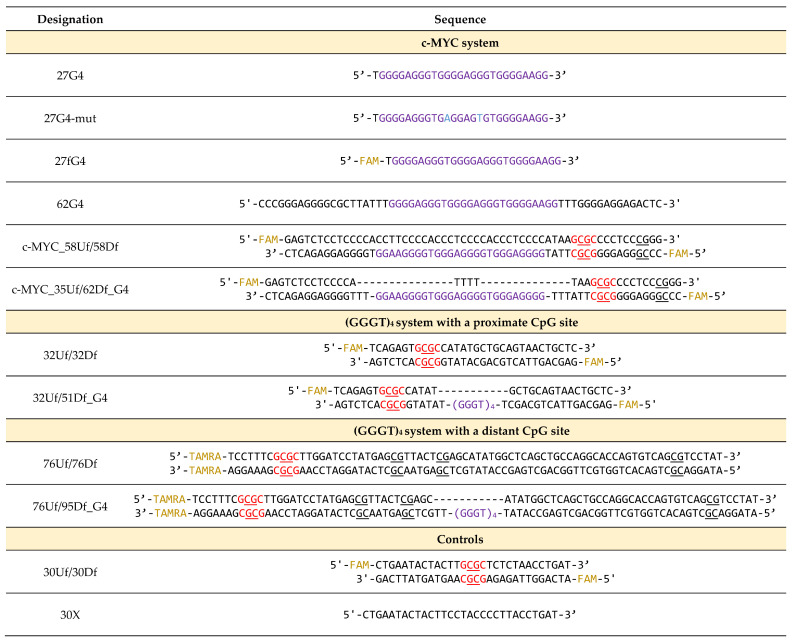

DNA model designations include oligonucleotide length, strand positions (indicated as “upper” (U) or “downstream” (D)); the presence of a fluorescent label (f); 5-carboxytetramethylrhodamine (TAMRA) or 6-carboxyfluorescein (FAM); and embedded G4 motif (G4). In the text, the “f” symbol has been omitted in oligonucleotide/duplex models that do not contain fluorescent labels. The CpG sites are underlined; GCGC Hin6I restriction sites are highlighted in red. FAM and TAMRA fluorophores are highlighted in yellow, G4 motifs are highlighted in purple, and substituted nucleotide residues are highlighted in blue.

## Data Availability

Data are contained within the article and Supplementary Materials.

## Supplementary Materials

The supporting information can be downloaded at: https://www.mdpi.com/article/10.3390/ijms25010045/s1.
