# Supplementary material for: Crosstalk between G-Quadruplexes and Dnmt3a-Mediated Methylation of the c-MYC Oncogene Promoter
Source: Int J Mol Sci. 2023 Dec 19;25(1):45. doi: 10.3390/ijms25010045 (PMC10779317; doi:10.3390/ijms25010045)
Supplement: Supplementary file 1 [file ijms-25-00045-s001.zip › ijms-2765628-supplementary.pdf]

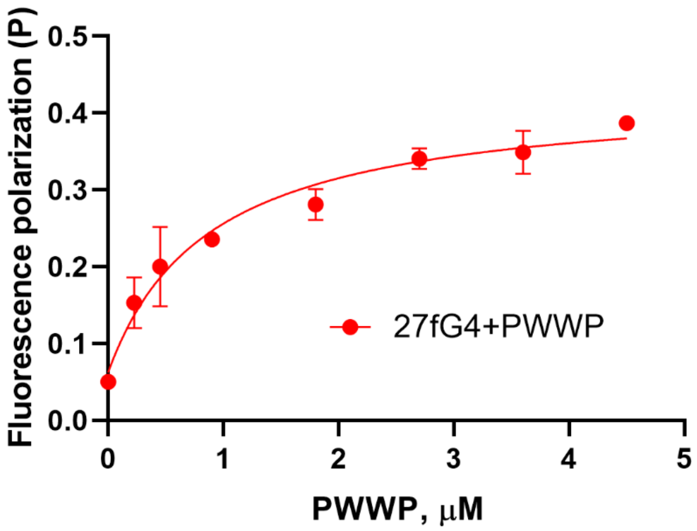

Figure S1. Binding curve of FAM-labeled 27fG4 to Dnmt3a PWWP domain derived from fluorescence polarization data. 60 nM 27fG4, 100 µM S-adenosyl-L-homocysteine, 20 mM HEPES-NaOH (pH 7.5), 100 mM KCl, 1 mM EDTA, 1 mM 1,4-dithiothreitol, 0 – 4.5 µM PWWP.
